# A Case Report of High-Grade Endometrial Stromal Sarcoma: A Rare Cause of Abnormal Uterine Bleeding in a Young Woman

**DOI:** 10.1155/2018/5906760

**Published:** 2018-11-28

**Authors:** Nuntasiri Eamudomkarn, Yuwadee Itarat, Pilaiwan Kleebkaow, Chumnan Kietpeerakool

**Affiliations:** Department of Obstetrics and Gynaecology, Faculty of Medicine, Khon Kaen University, Khon Kaen, Thailand

## Abstract

High-grade endometrial stromal sarcoma (HG-ESS) is a rare clinical entity, particularly among young women, and only few cases have been reported in the literature. Herein, we describe the case of a 21-year-old woman who presented with a four-month history of excessive bleeding per vagina. Endometrial curettage and cervical biopsy revealed a malignant round cell tumor suggestive of metastatic sarcoma of uterine origin. Computed tomography of the abdominopelvic region showed an enlarged uterus with diffused thickening throughout the entire endometrial cavity. Intraabdominal lymphadenopathy and ascites in the pelvic cavity were noted. The patient underwent total abdominal hysterectomy, bilateral salpingo-oophorectomy, resections of the enlarged pelvic nodes, omentectomy, and biopsy of the peritoneal nodules in the cul-de-sac. Histological examination revealed a tumor with a permeative growth pattern composed of uniformly high-grade round cells with brisk mitotic activity and extensive lymphovascular space invasion. Sections of the pelvic lymph nodes on both sides and the peritoneal nodule revealed multiple metastatic foci. Immunohistochemical studies showed positive diffuse staining for vimentin, CD 10, and cyclin D1. The pathological diagnosis was HG-ESS stage IIIC. The patient experienced rapid progression of the disease while receiving adjuvant treatment and succumbed eight months after the operation. HG-ESS is a rare cause of AUB in adolescents and young women but should be considered in the differential diagnosis.

## 1. Introduction

Abnormal uterine bleeding (AUB) is characterized as excessively heavy, prolonged, and/or frequent bleeding of uterine origin [1]. Approximately one-third of women will experience AUB during their lifetimes [2]. The most common cause of AUB in adolescents and young women is dysfunctional uterine bleeding [1]. Other possible etiologies of AUB in adolescents and young women include thyroid dysfunction, polycystic ovarian syndrome, bleeding associated with pregnancy (i.e., abortion or ectopic pregnancy), gestational trophoblastic disease, uterine leiomyoma, endometrial polyps, cervicitis, genital tract trauma, and bleeding disorders (i.e., von Willebrand disease, thrombocytopenia, or clotting factor deficiency) [1]. Cancers of the genital tract are an infrequent cause of AUB among adolescents and young women [1].

Endometrial stromal tumors (EST) are rare tumors of endometrial stromal origin and account for less than 2% of all uterine tumors [3, 4]. According to the 2014 World Health Organization (WHO) tumor classification system, EST can be classified into four categories: endometrial stromal nodule (ESN), low-grade endometrial stromal sarcoma (LG-ESS), high-grade endometrial stromal sarcoma (HG-ESS), and undifferentiated uterine sarcoma (UUS) [5].

HG-ESS is rare, particularly among young women [3]. An accumulation of reports of this rare clinical entity is, therefore, necessary to better understand its natural course. Herein, we report a case of HG-ESS in a young woman who presented with abnormal vaginal bleeding and a pelvic mass.

## 2. Case Report

A 21-year-old, G0P0 woman presented with a four-month history of excessive and prolonged bleeding per vagina, as well as a palpated mass at the lower abdomen that was rapidly increasing in size. Her past history was unremarkable. Two months before this visit, she had presented at the provincial hospital with severe anemic symptoms. She was found to have severe anemia and received a blood component transfusion. The excessive vaginal bleeding had persisted until one month prior to her presentation at our hospital. She had undergone endometrial curettage at the provincial hospital, and the pathological report indicted an atypical round cell tumor.

**Figure 1:**
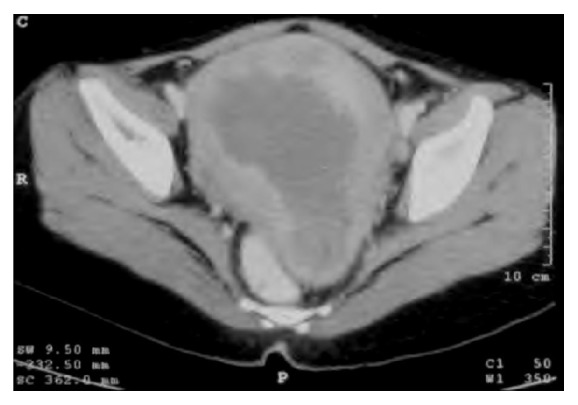
Computed tomography of the abdominopelvic region shows an enlarged well-defined border uterus with diffused enhancing thickening in the endometrial cavity.

Upon presentation at our hospital, she was pale and found to have a midline pelvic mass. Per vaginal examination revealed a 3 cm exophytic mass at the posterior lip of the uterine cervix and a 14 cm, firm uterine mass. Hematoxylin*-*eosin stained (H&E) slides of the endometrial specimen were reviewed. The sections showed a malignant round cell tumor with scattering foci and vascular architecture mixed with benign-looking endometrial glands. Computed tomography (CT) of the abdominopelvic region showed an enlarged, well-defined uterine border with diffused enhancing thickening in the endometrial cavity involving entire uterine body and cervix (Figure 1). The overall uterine size was 15.0x11.6x10.5 cm. Intra-abdominal lymphadenopathy and ascites in the pelvic cavity were noted. The liver, gallbladder, pancreas, spleen, bilateral kidneys, and bilateral adrenal glands appeared normal.

A biopsy of the cervical mass was performed to obtain tissue for further study of immunohistochemical (IHC) markers. The pathological examination showed a malignant small round cell tumor suggestive of metastatic sarcoma of uterine origin. Immunohistochemical studies showed negative staining for multi-cytokeratin (AE1/AE3), S-100 protein, CD 10, cyclin D1, caldesmon, myogenin, and desmin. The patient's tumor exhibited focal positive staining for smooth muscle actin. The preoperative differential diagnoses were HG-ESS and UUS.

At laparotomy, the uterus and obturator lymph nodes on both sides were enlarged. There were multiple nodules in the cul-de-sac. Neither the adnexae nor the omentum appeared remarkable. The surgical procedures included total abdominal hysterectomy, bilateral salpingo-oophorectomy, resections of enlarged pelvic nodes, omentectomy, and biopsy of peritoneal nodules in the cul-de-sac.

**Figure 2:**
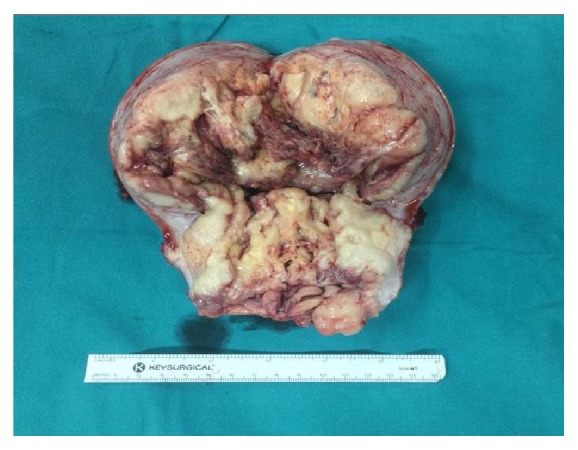
Gross examination of uterus reveals an enlarged uterus 13x13x7 cm with an intracavitary polypoid mass occupying the entire endometrial cavity and endocervical canal.

**Figure 3:**
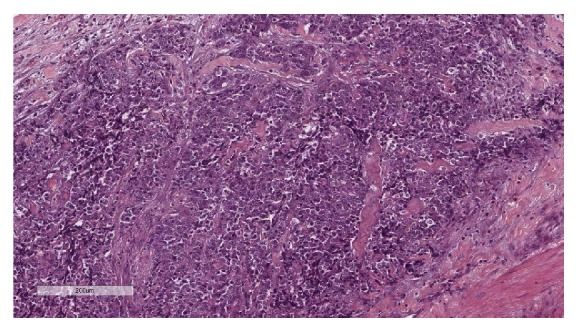
Microscopic examination reveals a small round cell tumor with high mitotic activity arranged in tight nests separated by a delicate capillary network (H&E staining; 20x).

**Figure 4:**
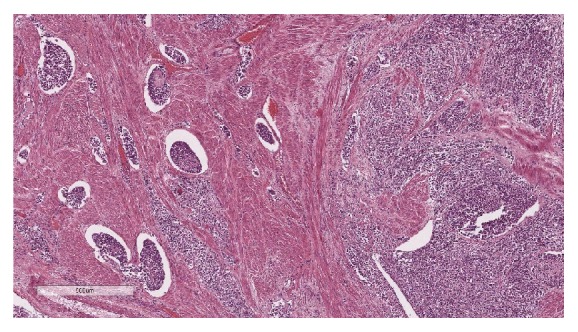
Microscopic examination reveals the presence of an extensive lymphovascular space invasion in the myometrium (H&E staining; 4x).

Macroscopically, the uterus weighed 714.67 grams and measured 13x13x7 cm with an intracavitary polypoid mass that occupied the entire endometrial cavity (Figure 2). The tumor had invaded the serosa of the uterus and ectocervix. Sectioning revealed a yellow cut surface with focal areas of hemorrhage. Histologically, the tumor exhibited a permeative growth pattern and was composed of uniformly high-grade round cells with brisk mitotic activity arranged in tight nests separated by a delicate capillary network (Figure 3). The tumor had invaded the uterine serosa, and there was extensive lymphovascular space invasion (LVSI) (Figure 4). Sections of the pelvic lymph nodes on both sides and peritoneal nodule revealed multiple metastatic foci. The omentum, both ovaries, and both fallopian tubes were histologically unremarkable. Additional IHC studies revealed positive diffuse staining for vimentin, CD 10, and cyclin D1. The tumor stained negative for desmin, estrogen receptors (ER), and progesterone receptors (PR). A diagnosis of HG-ESS stage IIIC was made based on these pathological findings.

The postoperative clinical course was uneventful. The patient was started on adjuvant chemotherapy consisting of Adriamycin (50 mg/m^2^) and Ifosfamide (5 g/m^2^) given every three weeks. After receiving four courses of this chemotherapy regimen, the patient refused further adjuvant chemotherapy due to her inability to tolerate the side-effects. Pelvic radiation was then administered thereafter. Despite undergoing radiation treatment, the patient experienced rapid disease progression and succumbed eight months after operation.

## 3. Discussion

Abnormal uterine bleeding is a common reason for adolescents and young women to present at healthcare facilities [2]. There are various causes of AUB in these patients, and cancer of the genital tract is relatively rare [1]. Possible genital tract cancers that cause AUB in adolescents and young women include endometrial cancer, vaginal rhabdomyosarcoma, and cervical adenocarcinoma [6–8]. In this report, we describe a case of HG-ESS in a 21-year-old woman who presented with abnormal vaginal bleeding and a pelvic mass.

High-grade endometrial stromal sarcoma is rare, constituting of less than 1% of uterine malignancies and less than 10% of uterine sarcomas [3, 4]. The common presenting symptoms of HG-ESS are abnormal vaginal bleeding, palpable masses, and pelvic pain [3]. The clinical presentations in our patient are generally consistent with those that have previously been reported except the patient's age. The average ages at HG-ESS diagnosis reported in the literature range from 40 years to 55 years [3]. The exceptional finding in our report, thus, is HG-ESS being diagnosed in a young patient.

Endometrial stromal sarcoma is a genetically heterogenous group of uterine sarcomas [9–11]. High-grade endometrial stromal sarcoma typically harbors t(10;17)(q22;p13) resulting in YWHAE-NUTM2A/B (previously known as YWHAE- FAM22) genetic fusion [5]. YWHAE-rearranged HG-ESS represents a molecularly and prognostically distinct type of ESS, which is associated with an aggressive natural course [10, 11]. The unique clinical behavior and treatment responses of this subset of ESS therefore merit pathologic evaluation to confirm the presence of the t(10;17)(q22;p13) translocation in cases of morphologically suspected HG-ESS [10]. Cyclin D1 has been proposed as a sensitive and specific diagnostic immunomarker for YWHAE-FAM22 ESS [9]. In a recent case series, the high-grade round cell component of all 12 YWHAE-FAM22 ESS exhibited diffuse moderate to strong nuclear cyclin D1 staining, and this diffuse positivity has not been seen in patients who harbored other genetic rearrangements [9]. The authors postulate the utility of cyclin D1 staining to indicate a provisional diagnosis of YWHAE-FAM22 ESS when encountering a uterine tumor in which the differential diagnosis of a histologically HG-ESS is considered [9]. In our patient, there was diffuse positive cyclin D1 staining, possibly indicating a provisional diagnosis of YWHAE-rearranged HG-ESS.

High-grade endometrial stromal sarcoma is also a group of tumors with heterogenous morphologic features [12]. Sciallis et al. [12] classified HG-ESS into three subgroups based on morphology: (I) tumors with a component that is similar to LG-ESS and transitions abruptly into a higher-grade component; (II) tumors composed exclusively of high-grade round cells with uniform nuclear features but with a permeative pattern of infiltration; and (III) tumors similar to the second group but with cytomorphology featuring enlarged round to ovoid cells with smooth nuclear membranes and distinct chromatin clearing but lacking prominent nucleoli. Lymphovascular space invasion is commonly noted in category III tumors [12]. In addition, the authors observed that most of the tumors in category III tested positive for YWHAE rearrangement [12]. Thus, cytomorphologic features on routine hematoxylin and eosin-stained sections may be helpful in classifying tumors with different molecular genetic changes.

According to the National Cancer Database, which is a nationwide, facility-based, comprehensive database established by the American Cancer Society and Commission on Cancer of the American College of Surgeons, survival in patients with HG-ESS remains poor [13]. The median overall survival was 19.9 months (95% CI, 17.1–22.1 months), and the five-year overall survival was only 32.6% (95% CI; 30.1-35.3%). Prognostic factors negatively associated with survival include patients' age, tumor size, omission of lymphadenectomy, pathologically positive resection margins, and distant or nodal metastasis [13]. Some of these poor prognostic factors were noted in our patient including large tumor size and metastases to the peritoneum and pelvic lymph nodes.

It is extremely rare for HG-ESS to be diagnosed in adolescents and young women, and only a few cases have been reported in the literature [14–16]. The prognosis of HG-ESS among this subset of women is generally poor due to the disease presenting at a later stage. Patients in some of the reported cases have succumbed to the extensive metastases of the disease in a relatively short time period after surgery [14, 15]. In our patient, the tumor rapidly progressed during adjuvant treatment and the patient passed away at eight months after the operation. The strength of this report is that the diagnosis of HG-ESS was confirmed by a panel of IHC staining. However, the definite molecular genetic change in our case was unknown.

## 4. Conclusion

We describe a case of HG-ESS stage IIIC diagnosed in a 21-year-old woman who presented with abnormal vaginal bleeding and a pelvic mass. Our report confirms the relatively poor prognosis of this subtype of ESS. Our patient experienced rapid disease progression during adjuvant treatment and passed away at eight months after surgery. This report highlights that HG-ESS is a rare cause of AUB in adolescents and young women but should be considered in the differential diagnosis.

## Data Availability

The data used to support the findings of this study are available from the corresponding author upon request.
